# Effect of Feed Additives on Productivity and *Campylobacter* spp. Loads in Broilers Reared under Free Range Conditions

**DOI:** 10.3389/fmicb.2017.00828

**Published:** 2017-05-12

**Authors:** Muriel Guyard-Nicodème, Adeline Huneau-Salaün, Fabrizio A. Tatone, Fabien Skiba, Maxime Quentin, Ségolène Quesne, Typhaine Poezevara, Marianne Chemaly

**Affiliations:** ^1^Hygiene and Quality of Poultry and Pork Products Unit, ANSES – Ploufragan-Plouzané Laboratory, Université Bretagne LoirePloufragan, France; ^2^Avian and Rabbit Epidemiology and Welfare Unit, ANSES – Ploufragan-Plouzané Laboratory, Université Bretagne LoirePloufragan, France; ^3^NUTRICIA – Route de Saint-SeverHaut-Mauco, France

**Keywords:** *Campylobacter*, control measure, feed additive, broiler, free-range

## Abstract

The poultry reservoir, especially broiler meat, is generally recognized as one of the most-important sources for human Campylobacteriosis. The measures to control *Campylobacter* targeted essentially the primary production level. The aim of this work was to evaluate the effectiveness of different treatments against natural *Campylobacter* colonization in a French experimental farm of free-range broilers during the whole rearing period. Five commercial products and a combination of two of them were tested and all the products were added to feed or to water at the dose recommended by the suppliers. *Campylobacter* loads in caeca and on carcasses of broilers at the slaughter were determined by culture methods. Natural contamination of the flock occurred at the end of the indoor rearing period between day 35 and day 42. At day 42, the multispecies probiotic added to the feed reduced the contamination of 0.55 log_10_ CFU/g (*p* = 0.02) but was not significant (*p* > 0.05) at the end of rearing at day 78. However, another treatment, a combination of a cation exchange clay-based product in feed and an organic acid mixture (formic acid, sodium formate, lactic acid, propionic acid) in water, led to a slight but significant reduction of 0.82 ± 0.25 log_10_ CFU/g (*p* = 0.02) compared to the control group at day 78. Testing this combination in field conditions in several flocks is needed to determine if it is biologically relevant and if it could be a valuable measure to reduce *Campylobacter* in broiler flocks.

## Introduction

Campylobacteriosis is the most commonly reported zoonosis in the EU since 2005 and 229 213 confirmed cases were reported in 2015 (European Food Safety Authority [EFSA], and European Centre for Disease Prevention and Control [ECDC], 2016). The infectious agent is *Campylobacter* spp. mainly *Campylobacter jejuni* (90%) and *Campylobacter coli* (10%), which cause an acute gastrointestinal infection in humans. The poultry reservoir, especially broiler meat, is generally recognized as one of the most-important source for human campylobacteriosis (European Food Safety Authority [EFSA], 2010b). In Europe, the mean prevalence of *Campylobacter* in primary poultry production is very high, up to 70% of broiler batches being contaminated with large differences ranging between 2 and 100% observed between countries (European Food Safety Authority [EFSA], 2010a). Moreover high numbers, up to 8 log CFU/g, of *Campylobacter* can be enumerated from broiler caecal contents (Hansson et al., 2010; Hue et al., 2010).

However, to date, no criteria have been established in the European legislation for *Campylobacter* spp. load in foodstuffs, and then a preventative approach is considered. Indeed, according to the study of Romero-Barrios et al. (2013), reducing *Campylobacter* spp. loads by 3-log_10_ in broilers’ gut would reduce the public health risk by at least 90%.

Evaluation of different potential interventions to prevent or to reduce *Campylobacter* colonization in broilers is still in progress, as there is no effective, reliable and practical strategy available so far. Some of them have been reviewed recently (Robyn et al., 2015; Sahin et al., 2015; Meunier et al., 2016a; Saint-Cyr et al., 2016a). Vaccination and the use of bacteriocins are not currently available, but they could represent promising measures in the future (Svetoch and Stern, 2010; Meunier et al., 2016a,b). Feed additives with non-antibiotic products such as probiotic bacteria, prebiotics, plant extracts or organic acids against *Campylobacter* colonization are still extensively studied. They give some promising results in experimental trials leading to at least 2 log_10_ CFU/g reduction or more in *Campylobacter* colonization for some of them (Skanseng et al., 2010; Ghareeb et al., 2012; Guyard-Nicodeme et al., 2016; Saint-Cyr et al., 2016b). In these studies testing feed additives, trials were generally performed using conventional production conditions: indoor rearing, broiler breeds, whole rearing (Hilmarsson et al., 2006; Thibodeau et al., 2015; Gracia et al., 2016; Guyard-Nicodeme et al., 2016), or shorter periods (Solis de Los Santos et al., 2008; Skanseng et al., 2010; Ghareeb et al., 2012). However, there is an increasing consumer interest in free-range poultry. In France, the free-range Label Rouge traditional poultry, accounted for 15% of the production and 60% of the consumption of whole broiler carcasses by French household (Salvat et al., 2017). Breeding conditions of free–range broilers are different from those of conventional production and vary according to the European Member States. According to the French Label Rouge specifications, slow growing breeds of broilers are reared with a lower breeding density indoor from 1 to 42 days old, and have access to an outdoor range from 6 weeks until depopulation at 81 days old at least. As for the conventional broilers, the free-range broiler flocks can be colonized by *Campylobacter* (Rivoal et al., 2005; Huneau-Salaün et al., 2007; Allen et al., 2011; Economou et al., 2015; Salvat et al., 2017). However, to the best of our knowledge the effect of feed additives against *Campylobacter* in free-range broilers has not been yet studied. The aim of this work was to evaluate the effectiveness of different additives against natural *Campylobacter* colonization in a French experimental farm of free-range broilers.

## Materials and Methods

### Ethics Statements

This study was carried out in an approved establishment for animal experimentation under the “Label Rouge” program specifications for rearing conditions by the aaa Direction Départementale de la Cohésion Sociale et de la Protection des Populations des Landes bbb (agreement number A-40-037-2). The protocol was designed and all practices were performed according to the 2010/63/EU regulation about animal welfare.

### Experimental Design

One day-old male chicks of strain T44 N x SA 51 (*n* = 1440), purchased from a commercial hatchery, were reared in the facilities of the Nutricia experimental farm (Benquet, Southwest of France). This facility is designed to replicate rearing conditions according to the Label Rouge program. At the hatchery, the birds were vaccinated against Marek’s Disease, Bronchitis infectious, Gumboro and Newcastle diseases and Coccidiosis. A booster vaccination for Bronchitis infectious was carried out at 21 days. Rearing temperature was held constant at 28°C during the first 3 days and then, it was gradually reduced until the fourth week to reach 20°C. This temperature was maintained until access to outdoor range after 42 days (according to the criteria of the Label Rouge specifications). A continuous light was applied during the first 48 h and was then reduced to 12 h per day.

Upon arrival, chicks were randomly allocated to one of the 36 pens (*n* = 40 chicks per pen). Six pens were randomly assigned to the control group, without any treatment (T1), and five pens were randomly assigned per treatment (T2–T7).

Five commercial products and a combination of two of them were tested and all the products were added to feed or to water at the dose recommended by the suppliers (**Table 1**). According to suppliers’ recommendations, treatments T3, T4, and T7 were distributed throughout the trial; treatments T2, T5, and T6 were distributed only from day 71 to day 78 (**Table 1**). Food and water were available *ad libitum*. Individual feeders and drinkers were displayed in each pen, avoiding feed contamination from one pen to the others. The birds were fed from day 1 to day 28 with a starter crumble, from day 29 to 49 with a grower mash and from day 50 to day 78 with a finisher mash (Supplementary Table S1). Formulation of the different diets were iso-caloric and iso-nitrogenous. Birds were slaughtered at D79 in a conventional slaughterhouse where skin sampling was performed (first broiler batch of the day).

**Table 1:** Tested products, doses, and period of distribution.

Treatment	Composition	Mode	Dose	Period
T1 (Control)	None	None	None	None
T2	Cation exchange clay based additive	Feed	0.25 kg/ton	D71–D78
T3	Multi-species Probiotic	Feed	1 kg/ton	D1–D78
T4	Prebiotic-like	Feed	1.25 kg/ton	D1–D78
T5	Organic acid mixture (formic acid, sodium formate, lactic acid, propionic acid)	Water	1 ml/L	D71–D78
T6	Clay based additive (T2) + Organic acid mixture (T5)	Feed + Water	0.25 kg/ton + 1 ml/L	D71–D78
T7	Fermented plant extract	Water	2 ml/L	D1–D78

### Sampling and Microbiological Analyzes

**FIGURE 1:**
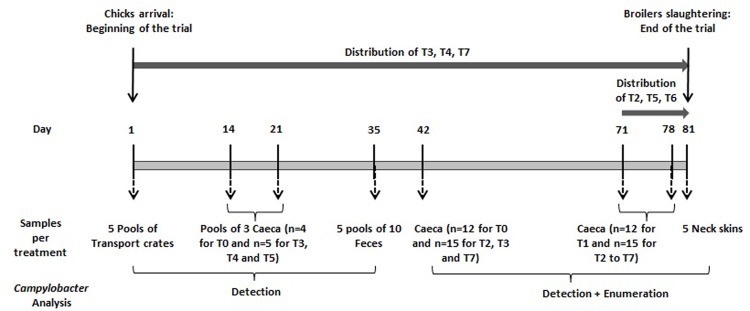
**Timeline of sampling and microbiological analyzes performed during the trial**.

Different types of samples were collected and analyzed during the course of the trial according to **Figure 1**. The sampling included the collection of cardboards at the bottom of the transport crates, fresh fecal material (pool of feces), caecal material (caeca or pool of caeca) after euthanasia (electronarcosis followed by bleeding) and neck skin samples. Until day 71, treatments T1, T2, T5, and T6 were not distributed, therefore, animals in these groups were confounded in a single control group called treatment T0.

The caecum was separated from the rest of the intestinal package through sterile scissors and placed in hermetically sealed plastic bags. Neck skin samples were collected from carcasses taken from the processing line after chilling at the slaughterhouse. After collection, samples were shipped in an insulated box to the ANSES laboratory (Ploufragan, Northwest, France) within 24 h with a cooler carrier (4°C). Samples were processed and analyzed upon arrival depending on the sample as following:

Absence of *Campylobacter* from cartons on the bottom of transport crates was assessed after enrichment according to part 1 of the ISO 10272 (Anonymous, 2006). Samples were weighed and diluted 1:10 (wt:vol) in Bolton broth and the mix was homogenated in a peristaltic homogenizer (AES, Bruz, France). For detection purposes, 10 ml of the homogenate was added to 90 ml of Bolton broth (Oxoid, Dardilly, France). The inoculated broth was then incubated under microaerophilic conditions for 4 h at 37°C and then for 44 ± 4 h at 41.5 ± 1°C.

Fecal materials were weighed and diluted 1:10 (wt:vol) in tryptone salt broth and the mix was homogenized in a peristaltic homogenizer (AES, Bruz, France). Presence or absence of *Campylobacter* was assessed after direct plating of the homogenate on mCCDA plates (modified Charcoal, Cefoperazone, Desoxycholate Agar, Oxoid, Dardilly, France), and incubation as above. Characteristic colonies were confirmed with optical microscopy analysis.

*Campylobacter* from caecal contents were recovered using direct plating and/or enumeration. Direct isolation of *Campylobacter* was assessed by direct seeding of the caecal content on mCCDA and 44 ± 4 h of incubation at 41.5 ± 1°C in a microaerophilic atmosphere (85% N_2_, 10% CO_2_, 5% O_2_). In order to assess *Campylobacter*’s counts, caecal contents were weighed, diluted in tryptone salt broth, and homogenized in a peristaltic homogenizer (AES, Bruz, France). Serial dilutions of the homogenate in tryptone salt broth, were plated on selective mCDDA plates and enumeration was assessed after incubation as above. The threshold for enumeration was 2 × 10^2^ CFU/g (2.3 log_10_ CFU/g) of caecal content.

For the neck skin samples, detection after enrichment and enumeration of *Campylobacter* was performed according to part 1 and 2 of the ISO 10272 (Anonymous, 2006) respectively. Samples were weighed and diluted 1:10 (wt:vol) in tryptone salt broth and the mix was homogenated in a peristaltic homogenizer (AES, Bruz, France). For detection purposes, 10 ml of the homogenized was added to 90 ml of Bolton broth (Oxoid, Dardilly, France). The inoculated broth was then incubated under microaerophilic conditions for 4 h at 37°C and then for 44 ± 4 h at 41.5 ± 1°C. The culture in Bolton broth was subsequently plated onto mCCDA and Butzler agar (Virion N°2) (Oxoid, Dardilly, France) and incubated for 44 ± 4 h at 41.5 ± 1°C. Characteristic colonies were confirmed with optical microscopy analysis. For the quantification, *Campylobacter* were enumerated by plating 1 ml of the homogenate onto three plates of mCCDA. Tenfold serial dilutions of the homogenate in tryptone salt broth were also prepared and plated onto mCCDA plates. All plates were incubated under microaerophilic conditions for 44 ± 4 h at 41.5 ± 1°C. The threshold for enumeration was 10 CFU/g (1 log_10_ CFU/g) of neck skin.

### Performance

Animal weights were recorded individually at days 14, 28, 53, 72, 79 and at the slaughterhouse. The record of food consumption took place weekly and at the day of weighing. Daily consumption, daily gain and feed conversion ratio (FCR) were calculated for 3 periods: from D1 to D28, D29 to D72 and D73 to slaughter. Mortality was recorded daily and dead animals were weighted individually.

### Statistical Analysis

*Campylobacter* loads in caeca, weight and feed consumption were analyzed using an ANOVA model including the treatment as a fixed effect and the pen as a random effect; *post hoc* tests were carried out for mean comparisons (Tukey test, *P* < 0.05). For comparison of *Campylobacter* loads on neck skin, the ANOVA model only included the treatment as a fixed effect.

## Results

### Effect of Treatments on *Campylobacter* Colonization

No *Campylobacter* was detected on chick transport crate. From day 1 to day 35, *Campylobacter* was not recovered from the samples, whatever the treatment was (data not shown). *Campylobacter* was detected in samples from day 42 onward. Therefore, natural contamination of the flock occurred between day 35 and day 42, and the treatments T3, T4, and T7 did not prevent colonization of the broilers.

At day 42, enumeration of *Campylobacter* in caecal contents was performed to determine if the treatments T3, T4, and T7 distributed from day 1 impacted *Campylobacter* loads in caeca, compared to the control group T0 (constituted of samples from the T1, T2, T5, and T6 groups). As shown in **Table 2**, broilers that received the treatment T3 (Multispecies probiotic) had significantly lower *Campylobacter* counts than the control group (*P* = 0.02). However, the observed mean reduction was less than 1 log_10_ CFU/g. Treatments T4 (prebiotic-like) and T7 (fermented product) did not lead to a significant reduction compared to the control group.

**Table 2:** Effect of dietary treatment on *Campylobacter* counts (Log_10_CFU/g, mean ± standard deviation) in the caeca of broilers at 42, 71, and 78 days of age.

	D42			D71			D78		
Treatment	*n*	log_10_CFU/g (Mean ± SD)	CI_95%_	*n*	log_10_CFU/g (Mean ± SD)	CI_95%_	*n*	log_10_CFU/g (Mean ± SD)	CI_95%_
T0	12	8.43 ± 0.53^a^	8.15-8.72	-	-	-	-	-	
T1	-	-	-	10	6.83 ± 1.00^a,b^	6.24–7.42	12	6.71 ± 0.67^a^	6.34–7.07
T2	-	-	-	15	6.79 ± 1.11^a,b^	6.25–7.34	15	6.51 ± 0.75^a,b^	6.14–6.88
T3	15	7.88 ± 0.43^b^	7.66–8.09	15	6.70 ± 0.81^a,b^	6.30–7.10	15	6.16 ± 0.85^a,b^	5.75–6.58
T4	15	7.96 ± 0.60^a,b^	7.67–8.25	15	6.92 ± 0.80^a^	6.53–7.31	15	6.22 ± 1.16^a,b^	5.65–6.79
T5	-	-	-	15	6.69 ± 0.69^a,b^	6.35–7.02	15	6.38 ± 1.00^a,b^	5.89–6.87
T6	-	-	-	14	6.86 ± 0.98^b^	6.63–7.35	15	5.88 ± 0.96^b^	5.41–6.35
T7	15	8.11 ± 0.59^a,b^	7.82–8.40	15	6.10 ± 1.29^a,b^	5.47–6.74	15	5.71 ± 1.82^a,b^	4.82–6.61

*At 42 days, only treatment T3, T4, and T7 were distributed, so T1, T2, T5, and T6 constituted the control group T0. At D71 and D78, T1 corresponded to the control group. ^a,b^Means within the same column with different superscript are significantly different (*p* < 0.05).*

A second sampling was carried out at day 71 to determine the contamination levels in the groups T2, T5, and T6 before application of the treatments and to check the effect of the treatments T3, T4, and T7 compared to the control group T1. At day 71, the contamination level was not significantly different in the groups T2, T5, and T6 before application of treatments compared to the control group T1. *Campylobacter* loads in the three treated groups T3, T4, and T7 were not significantly different than the one observed in the control group T1.

Otherwise, the mean *Campylobacter* loads in all treatment decreased from 8.08 log_10_ CFU/g (CI_95%_ [7.93–8.22]) at D42 to 6.69 log_10_ CFU/g (CI_95%_ [6.50–6.88]) at D71. The decrease was also observed in the control treatment (8.43 log_10_ CFU/g (CI_95%_ [8.15–8.72]) at D42 vs. 6.83 log_10_ CFU/g (CI_95%_ [6.24–7.42]) at D71).

Results at day 78 revealed that the three groups receiving a treatment since the beginning of the trial (T3, T4, and T7) did not show a significant reduction of the colonization compared to the control group. Among the three groups receiving a product only during the last week of rearing (T2, T5, and T6), only T6 (combination T2 + T5: a clay-based product in feed, and an organic acid mixture in water, respectively) showed a significant reduction estimated at 0.82 ± 0.25 log_10_ CFU/g (*p* = 0.02) compared to the control group.

### Effect of Treatments on *Campylobacter* Contamination of Carcasses (Neck Skin)

At slaughter, carcasses from treatment T6 showed a slight but significant (*p* = 0.01) reduction estimated at 0.68 ± 0.24 log_10_ CFU/g in *Campylobacter* counts on neck skin samples compared to the control group T1 (**Table 3**). No other significant difference was observed between the control group and the other treatments. Nevertheless, these results need to be confirmed using a higher number of samples, as only five carcasses per group were sampled in this study.

**Table 3:** *Campylobacter* loads (log_10_ CFU/g) on neck skin at D81.

Treatment	*n*	log_10_CFU/g (Mean ± SD)	CI_95%_
T1	5	1.70 ± 0.41^a^	1.20–2.21
T2	5	1.62 ± 0.28^ab^	1.28–1.97
T3	5	1.46 ± 0.31^ab^	1.08–1.84
T4	5	1.03 ± 0.65^ab^	0.22–1.84
T5	5	1.31 ± 0.34^ab^	0.89–1.74
T6	5	1.02 ± 0.16^b^	0.83–1.21
T7	5	1.09 ± 0.28^ab^	0.73–1.45

*^a,b^Means within the same column with different superscript are significantly different (p < 0.01).*

### Effect of Treatments on Growth Performance

Broilers from T3, T4, and T7 showed a higher daily weight gain in comparison with the ones from treatments with no additive during the first rearing period but their FCR was not significantly improved (**Table 4**). Over the whole rearing period no constant effect of the treatments were observed on daily feed consumption, daily weight gain and FCR. The mean mortality rates varied from 0.07% in T6 (1/240) to 3.8% (7/200) in T1 with no significant difference between treatments (data not shown).

**Table 4:** Daily feed consumption (g), daily body weight gain (g) and feed conversion ratio (FCR) from D1 to D28, from D29 to D72, from D73 to slaughter and over the whole rearing period according to the treatments (Least Squares Mean ± Standard Error).

	D0–D28						D29–D72						D73–D79						D0–D79					
	Cons		Daily gain		F.C.R		Cons		Daily gain		F.C.R		Cons		Daily gain		F.C.R		Cons		Daily gain		F.C.R	
T1	37.3^ab^	0.6	20.0^a^	0.3	1.86	0.02	114.4	1.7	37.9	0.6	3.03^ab^	0.04	169.1	5.6	43.3	2.2	3.97	0.26	91.9	1.4	32.1	0.4	2.87	0.04
T2	37.2^ab^	0.6	20.3^a^	0.3	1.84	0.02	115.0	1.9	38.0	0.6	3.03^ab^	0.04	171.8	6.1	43.5	2.4	3.98	0.28	92.5	1.6	32.2	0.5	2.87	0.04
T3	40.0^b^	0.6	21.9^bc^	0.3	1.82	0.02	115.7	1.9	37.8	0.6	3.06^ab^	0.04	180.4	6.1	43.3	2.4	4.17	0.28	94.6	1.6	32.6	0.5	2.90	0.04
T4	39.6^b^	0.6	22.4^c^	0.3	1.77	0.02	117.1	1.9	37.1	0.6	3.16^b^	0.04	172.5	6.1	46.4	2.4	3.76	0.28	94.5	1.6	32.7	0.5	2.90	0.04
T5	37.4^ab^	0.6	20.2^a^	0.3	1.86	0.02	112.7	1.9	37.6	0.6	3.00^ab^	0.04	175.7	6.1	43.5	2.4	4.05	0.28	91.6	1.6	31.9	0.5	2.87	0.04
T6	36.7^a^	0.6	19.9^a^	0.3	1.84	0.02	113.7	1.9	38.7	0.6	2.94^a^	0.04	170.1	6.1	40.2	2.4	4.42	0.28	91.4	1.6	32.2	0.5	2.84	0.04
T7	37.9^ab^	0.6	20.6^ab^	0.3	1.84	0.02	113.9	1.9	38.7	0.6	2.95^a^	0.04	176.9	6.1	45.6	2.4	3.93	0.28	92.5	1.6	32.9	0.5	2.81	0.04

*^a,b^Means within the same column with different superscript are significantly different (p < 0.05).*

## Discussion

Animal welfare is an increasing important issue for the consumers (Napolitano et al., 2010) and therefore there is a growing interest for free range and/or organically ranged broilers. However, the free-range rearing conditions bring together several of the known risk factors favoring *Campylobacter* colonization in broilers with for example the contact of the birds with an open environment and the age of the birds at slaughter (Huneau-Salaün et al., 2007; Newell et al., 2011). In France a representative study conducted in 2008 demonstrated that prevalence of *Campylobacter* in caecal contents of slaughtered batches was 100.0% for the Label chickens compared to 69.7% for the standard chickens (Hue et al., 2010).

During this study, broilers were naturally colonized by *Campylobacter* at the end of the indoor rearing period between day 35 and day 42. These results are in agreement with those of Huneau-Salaün et al. (2007) who reported that 71.2% of French free-range flocks are positive for *Campylobacter* at the end of the indoor rearing period. However, in some cases, broilers become colonized by *Campylobacter* after 6 weeks of rearing inside the building, when they can have access to the outdoor range (Rivoal et al., 2005).

Developing a control strategy against *Campylobacter* in the primary production is needed. Finding an effective product to be added to feed or water among the already marketed products could be a rapid solution. The tested products of this study were chosen based on a claimed activity, such as reducing pathogen, limiting bacterial growth or digestive pathogens, and/or improving immune functions. Five products and a combination of two of them were evaluated in the same trial. They were added according to the manufacturer’s conditions. None of the three treatments (T3, T4, and T7) used from day 1 was able to prevent *Campylobacter* colonization detected at day 42. Similar results were observed in previous works testing several feed additives in experimental facilities with artificial *Campylobacter* contamination on fast-growing broilers (Gracia et al., 2016; Guyard-Nicodeme et al., 2016; Saint-Cyr et al., 2016b). Moreover, no treatment using single product (T2, T3, T4, T5, and T7) led to a significant reduction of *Campylobacter* in caeca, compared to the control group at the end of the rearing period. Treatment T2 (clay-based product) was previously tested in experimental facilities with artificial *Campylobacter* challenge and a mean reduction of 2.5 log_10_ CFU/g was observed in fast-growing birds after 36 days of rearing, although it failed to reduce the pathogen in slow growing birds in the same conditions (Guyard-Nicodème et al., 2014). It could be hypothesized that product efficacy could be impacted by the broiler breeds. However, Gormley et al. (2014) demonstrated that *Campylobacter* colonization is not affected by the broiler breeds (fast or slow growing breeds).

On the contrary, treatment T6, using the combination of the clay-based product (T2) in feed and an organic acid mixture in water (T5), led to a significant reduction of *Campylobacter* spp. counts in the caeca, and this reduction was also observed on neck skin at the slaughterhouse. Reduction in the caeca was low, as less than 1 log_10_ CFU/g (0.82 ± 0.25 log10 CFU/g) was observed. Several previous studies presented results of feed or water additives leading to a reduction of *Campylobacter* colonization higher than 2 log_10_ CFU/g but they were performed in experimental facilities with an artificial *Campylobacter* challenge (Nishiyama et al., 2014; Arsi et al., 2015; Gracia et al., 2016; Guyard-Nicodeme et al., 2016; Saint-Cyr et al., 2016b). However, these controlled conditions cannot reflect the field conditions, especially free-range conditions exposed to multiple sources of contaminations and contaminated with genetically diverse *Campylobacter* isolates (Rivoal et al., 2005). Anyway, the reduction of *Campylobacter* obtained with T6 was less than 1 log_10_ CFU/g at the flock, and the slight reduction observed at the slaughterhouse, could have an impact on public health. Indeed, reduced colonization in caecal contents of flocks by 1 log_10_ unit could reduce the number of campylobacteriosis cases from 48 to 83% (Romero-Barrios et al., 2013). Moreover this combination was used only the last week of rearing and had no impact on performance parameters. Therefore, these results need to be confirmed in other field trials using several other flocks to determine if it could be applied as an efficient control measure.

## Author Contributions

MG-N, AH-S, MQ, and MC contributed to the conception and design of the study. FT, SQ, and TP performed the experiments and MQ supervised the trial in the experimental farm. MG-N, AH-S, and FT analyzed the results and wrote the paper. FS and MC critically analyzed and revised the manuscript.

## Conflict of Interest Statement

The authors declare that the research was conducted in the absence of any commercial or financial relationships that could be construed as a potential conflict of interest.
