# Supplementary material for: Effect of Feed Additives on Productivity and Campylobacter spp. Loads in Broilers Reared under Free Range Conditions
Source: Front Microbiol. 2017 May 12;8:828. doi: 10.3389/fmicb.2017.00828 (PMC5427123; doi:10.3389/fmicb.2017.00828)
Supplement: Supplementary file 1 [file Table_1.DOCX]

Table S1. Ingredient and nutritional composition of the experimental diet used for the control group. The same composition of the diet was used for the groups treated with feed additives, except that a part of corn corresponding to the quantity of feed additive was removed.

|  | **Starter (D0-D28)** | **Grower (D29-D72)** | **Finisher (D73-D79)** |
| --- | --- | --- | --- |
| **Ingredients (%)** |  |  |  |
| Corn | 35.840 | 50.015 | 50.155 |
| Wheat | 20.500 | 15.400 | 21.000 |
| Wheat bran | 1.500 | 3.250 | --- |
| Corn DDGS | 5.000 | 6.000 | 10.000 |
| Sunflower meal | 4.000 | 5.000 | 7.000 |
| Soybean meal | 21.250 | 10.900 | 1.800 |
| Rapeseed meal | 5.000 | 5.000 |  |
| Soybean oil | 0.250 | 0.250 | --- |
| Starter premix | 0.200 | --- | --- |
| Grower premix | --- | 0.100 | --- |
| Finisher premix | --- | 0.200 | 0.200 |
| Calcium carbonate | 1.125 | 0.975 | 1.075 |
| Dicalcium phosphate | 1.025 | 0.625 | 0.400 |
| Sodium chloride | 0.255 | 0.330 | 0.285 |
| Sodium sulfate | 0.100 | --- | --- |
| Lysine | 0.534 | 0.492 | 0.655 |
| Methionine | 0.278 | 0.133 | 0.082 |
| Threonine | 0.020 | --- | 0.020 |
| Xylanase | 0.015 | 0.015 | 0.015 |
| Phytase | 0.015 | 0.015 | 0.017 |
| Corn gluten | --- | 1.250 | 2.000 |
|  |  |  |  |
| **Nutriments** |  |  |  |
| apparent metabolizable energy (kcal/kg) | 2,841.040 | 2,918.376 | 3,002.566 |
| Proteins (%) | 20.000 | 17.000 | 15.300 |
| Crude fat (%) | 2.686 | 3.055 | 3.189 |
| Crude fiber (%) | 4.739 | 4.613 | 4.512 |
| Crude ash (%) | 5.515 | 6.685 | 4.126 |
| Starch (%) | 39.700 | 44.211 | 47.051 |
| Phosphore (%) | 0.606 | 0.525 | 0.471 |
| Chlorine (%) | 0.235 | 0.285 | 0.271 |
| Sodium (%) | 0.160 | 0.160 | 0.160 |
| Digestible Lysine (%) | 1.090 | 0.850 | 0.750 |
| Digestible Methionine (%) | 0.531 | 0.387 | 0.340 |
| Digestible Methionine + Cysteine (%) | 0.820 | 0.650 | 0.594 |
| Digestible Threonine (%) | 0.680 | 0.555 | 0.501 |
|  |  |  |  |
